# The metastatic tumor antigen 1-transglutaminase-2 pathway is involved in self-limitation of monosodium urate crystal-induced inflammation by upregulating TGF-β1

**DOI:** 10.1186/s13075-015-0592-7

**Published:** 2015-03-19

**Authors:** Jia-Hau Yen, Ling-Chung Lin, Meng-Chi Chen, Zsolt Sarang, Pui-Ying Leong, I-Chang Chang, Jeng-Dong Hsu, Jiunn-Horng Chen, Yu-Fan Hsieh, Anna Pallai, Krisztina Köröskényi, Zsuzsa Szondy, Gregory J Tsay

**Affiliations:** Institute of Microbiology and Immunology, Chung Shan Medical University, Taichung, Taiwan; Division of Dental Biochemistry, Department of Biochemistry and Molecular Biology, Research Center of Molecular Medicine, University of Debrecen, Debrecen, Hungary; Department of Internal Medicine, Chung Shan Medical University Hospital, No. 110, Section 1, Chien-Kuo N. Road, Taichung, Taiwan; Department of Orthopedic Surgery, Chung Shan Medical University Hospital, Taichung, Taiwan; Department of Pathology, Chung Shan Medical University Hospital, Taichung, Taiwan; Division of Immunology and Rheumatology, Department of Internal Medicine, China Medical University Hospital, Taichung, Taiwan

## Abstract

**Introduction:**

Transglutaminase 2 (TG2), a protein crosslinking enzyme with multiple biochemical functions, has been connected to various inflammatory processes. In this study, the involvement of TG2 in monosodium urate (MSU) crystal-induced inflammation was studied.

**Methods:**

Immunohistochemistry, reverse transcription-polymerase chain reaction (RT-PCR) were performed to detect TG2 expression in synovial fluid mononuclear cells (SFMCs) and synovial tissue from patients with gouty arthritis. MSU crystal-exposed RAW264.7 mouse macrophages were analyzed for interleukin-1β (IL-1β), tumor necrosis factor-α (TNF-α), transforming growth factor β1 (TGF-β1) and TG2 expression by RT-PCR and enzyme-linked immunosorbent assay (ELISA). TG2 small interfering (si)-RNA-mediated silencing and overexpression in RAW264.7 cells were used to evaluate the involvement of TG2 in resolving MSU crystal-induced inflammation. The role of metastatic tumor antigen 1 (MTA1), a master chromatin modifier, was investigated by MTA1 si-RNA-mediated knockdown. In addition, the inflammatory responses were followed in wild type and TG2 null mice after being challenged with MSU crystals in an in vivo peritonitis model.

**Results:**

TG2 expression was up-regulated in the synovium tissue and SFMCs from patients with gouty arthritis. The levels of MTA1, TG2, TGF-β1, IL-1β and TNF-α mRNAs were consistently increased in MSU crystal-stimulated RAW264.7 cells. si-MTA1 impaired the basal, as well as the MSU crystal-induced expression of TG2 and TGF-β1, but increased that of IL-1β and TNF-α. TG2 overexpression dramatically suppressed MSU crystal-induced IL-1β and TNF-α, but significantly enhanced the TGF-β1 production. Neutralizing TGF-β antibodies or inhibition of the crosslinking activity of TG2 attenuated these effects. On the contrary, loss of TG2 resulted in a reduced TGF-β, but in an increased IL-1β and TNF-α production in MSU crystal-stimulated RAW264.7 cells and mouse embryonic fibroblasts (MEFs). MSU crystal-stimulated IL-1β production was Janus kinase 2 (JAK2)-signaling dependent and TG2-induced TGF-β suppressed the activity of it. Finally, TG2-deficient mice exhibited hyper inflammatory responses after being challenged with MSU crystals in an *in vivo* peritonitis model.

**Conclusions:**

These findings reveal an inherent regulatory role of the MTA1-TG2 pathway in the self-limitation of MSU crystal-induced inflammation via positively regulating the levels of active TGF-β1 in macrophages that opposes the MSU crystal-induced JAK2-dependent pro-inflammatory cytokine formation.

**Electronic supplementary material:**

The online version of this article (doi:10.1186/s13075-015-0592-7) contains supplementary material, which is available to authorized users.

## Introduction

Gouty arthritis (GA) is a characteristically intense acute inflammatory reaction, which is initiated by precipitation of monosodium urate (MSU) crystals [1]. An attack of is a paradigm for acute sterile self-limited inflammation that is triggered by interactions between MSU microcrystals and the local tissue environment [2]. Our current understanding suggests that resident cells with such tissues, such as resident macrophages or monocytes, react first to crystal deposition by uptake of crystals through phagocytosis [3]. Through a series of steps that are still not well-understood, these phagocytes activate the NLRP3 inflammasome, resulting in the activation of caspase-1 and processing and secretion of IL-1β [4]. IL-1β then acts primarily on non bone-marrow-derived cells, such as capillary endothelial cells, by triggering MyD88-dependent events [5,6]. Experimental data on various knockout mice [7] and the rapid clinical response of patients with acute GA to IL-1 inhibition [8] validate the concept that IL-1 plays a key role in the initiation of gouty inflammation.

Besides IL-1β, uptake of MSU crystals by phagocytes also triggers the production of the pro-inflammatory cytokine IL-6 [9] and TNF-α [10], as well as that of the anti-inflammatory transforming growth factor β (TGF-β1) [11]. It was suggested that TGF-β1, a powerful inhibitor of inflammation [12], is involved in the resolution of gouty inflammation in experiments, in which administration of recombinant TGF-β1 attenuated MSU crystal-induced inflammation in an *in vivo* air-pouch model [13], and because of the observation that upregulation of TGF-β1 is strongly associated with the rapid resolution of acute GA [14,15]. TGF-β1 significantly inhibits leukocyte infiltration into air pouches injected with MSU crystals [13], and suppresses monocyte pro-inflammatory cytokine release in response to MSU crystals, endothelial cell activation in response to monocyte-derived cytokines, and macrophage release of TNF-α. In addition, this growth factor may contribute to fibroblast proliferation and the physical encasing of crystals away from contact with leukocytes [16]. TGF-β1 is normally secreted as part of a protein complex, which associates on the cell surface with α_v_-containing integrins [17,18]. TGF-β1 can be activated by the proteolysis of latency-associated peptide-β1 leading to the release of free TGF-β1 [19]. Transglutaminase 2 (TG2), which acts as a co-receptor for α_v_β_1_ and β_3_ integrins [20], has been suggested to contribute to TGF-β1 activation by bringing latency-associated peptide-β1 and the proteolytic activity on the macrophage cell surface into close proximity [17,21].

TG2 is a unique member of an enzyme family, because in addition to its protein cross-linking activity, it can act also as a G protein or co-receptor for the β_1_ and β_3_ integrins [22,23]. TG2 in macrophages has been shown to require the metastatic tumor antigen 1 (MTA1), a ubiquitously expressed chromatin modifier that regulates transcription of its targets by modifying the acetylation status of the target chromatin and the consequent co-factor accessibility to the target DNA, both for its basal and lipopolysaccharide-induced expression [24]. TG2, found both extracellularly at the cell surface in association with the extracellular matrix, and intracellularly in membrane-associated as well as in cytosolic forms, has been implicated in a variety of cellular processes including phagocytosis of apoptotic cells by macrophages [25,26]. By being involved in the clearance of apoptotic neutrophils by macrophages, TG2 has been suggested to limit GA via promoting the resolution phase of inflammation [27].

In the present paper we propose that TG2 might limit GA also by participating in the initiation phase of the disease, in which TG2 expressed at higher levels on the surface of MSU crystal-exposed macrophages enhances MSU crystal-induced TGF-β1 production which, in turn, attenuates MSU crystal-induced JAK2-dependent IL-1β release.

## Methods

### Reagents

MSU crystals were prepared as described previously [5]. Briefly, 5 mg/ml uric acid (Sigma, St Louis, MO, USA) was dissolved in 0.1 M Borate buffer (Ph 8.5) and added NaOH to keep the pH between 8.5 and 9.0. The solution was cleaned of any undissolved solid using a 0.45-m filter. The crystals came out of solution within 24 hours and then the precipitation continued for about a week. Finally, the crystals were washed twice in the centrifuge with absolute alcohol and then once with acetone. Needle-like crystals were recovered and were suspended in sterile saline. Recombinant mouse TG2 and mouse IgG isotype control were purchased from R&D Systems (Minneapolis, MN, USA).

### Sample collection

Synovial fluid was collected from the swollen knee joints of 16 patients with GA in acute phase, who were receiving uricosuric agents including allopurinol and benzbromarone, 5 patients with rheumatoid arthritis and 20 patients with osteoarthritis. The synovial fluid white blood cell (WBC) count of the 16 patients with gouty arthritis were between 2.5 and 61.8 K/uL (mean 25.2 K/uL). Synovial tissue (ST) specimens were collected from patients with GA at knee arthroscopy, from healthy individuals who had experienced traffic accidents*,* and patients with osteoarthritis during total knee replacement. Needle-shaped MSU crystals were detected intracellularly and extracellularly in synovial fluid by polarized light microscopy. All subjects gave written informed consent, and the study has been approved by the Institutional Review Board of the Chung Shan Medical University Hospital. Diagnosis of all patients with GA [28], osteoarthritis [29] or rheumatoid arthritis [30] conformed to the current American College of Rheumatology criteria.

### Cell culture and isolation

The murine macrophage cell line RAW264.7 was purchased from Bioresource Collection and Research Center (Hsinchu, Taiwan). Mouse embryonic fibroblast deficient in TG2 (TG2-KO MEF cells) and wild type (WT) MEF cells were generous gifts provided by Dr. Mauro Piacentini. All cells were grown in DMEM (Gibco/BRL, Grand Island, NY, USA) containing 10% fetal bovine serum (Biological Industries, Kibbutz Beit Haemek, Israel) and 1% Pen-Step Ampho. Solutionb (Biological Industries). Monocyte-derived macrophages (MDMs) were isolated from peripheral blood mononuclear cells (PBMCs) by plastic adherence. Phenotypical and functional characterization of MDMs was performed after 6 to 7 days. Synovial fluid mononuclear cells (SFMCs) were isolated from 16 patients with GA, 5 patients with rheumatoid arthritis and 20 patients with osteoarthritis using Ficoll-Hypaque gradient (Sigma-Aldrich). PBMCs isolated from five healthy donors served as normal controls.

### Treatment with MSU crystals

Treatment with MSU crystals was performed at 37°C at concentrations of 1 mg/ml for RAW264.7 cells, PBMCs and MDMs, and 2 mg/ml for MEF cells for the indicated time periods, because we found that 1 mg/ml of MSU crystals is sufficient to induce cytokine production in RAW264.7 cells, PBMCs and MDMs, but not enough to do so in MEF cells. In the initial experiments, a significant increase in IL-1β and TGF-β1 mRNA expression was evident as early as 30 minutes after MSU crystals challenge, which reached its peak at 4 h. That is why many of the experiments were carried out at this time point. None of the doses of MSU crystals used in the experiments induced cell death within 48 h, as examined by Annexin V-FITC and PI staining(AbD Serotec, Oxford, UK) followed by FACSCAN laser flow cytometry analysis (Becton Dickenson, San Jose, CA, USA).

### Immunohistochemistry

The synovial tissues were embedded in paraffin and cut into sections. The resulting specimens were treated with 3% H_2_O_2_ for 10 minutes to block endogenous peroxidase activity and incubated with normal horse serum (Santa Cruz Biotechnology, Santa Cruz, CA, USA). The sections were incubated with primary antibodies against TG2 or isotype control IgG (Sigma) at 4°C for 24 h and then detected using Histofine simple stain MAX PO (MULTI or G; Nichirei, Tokyo, Japan). Subsequently color was developed using diaminobenzidine (Dako, Carpinteria, CA, USA), and the sections were counterstained with hematoxylin (Vector Laboratories, Burlingame, CA, USA).

### Reverse transcription-polymerase chain reaction (RT-PCR)

Total RNA was isolated from cells by using the Trizol reagent protocol (Sigma). cDNA was synthesised using M-MLV Reverse Transcriptase (Promega, Madison, WI, USA). PCR was performed using TaKaRa Taq™ Polymerase. Each sample was tested in triplicate and normalized with glyceraldehyde-3-phosphate dehydrogenase (GAPDH) as an endogenous control. Sequences for the PCR primers are listed in Additional file 1.

### Cytokine production analysis

Cells were treated with MSU crystals or left untreated for 36 h. After treatment, the concentrations of IL-1β, TNF-α and TGF-β1 reaching its peak secretion in the culture medium were determined by ELISA according to the manufacturer’s instructions (Invitrogen, Carlsbad, CA, USA).

### Western blot analysis

The whole-cell protein lysates were size-fractionated by 10% SDS-polyacrylamide gels, and then transferred into polyvinylidene fluoride (PVDF) membranes (Millipore, Billerica, MA, USA). Membranes were blotted overnight at 4°C with one of the following primary antibodies: rabbit anti-TG2 (Thermo Fisher Scientific Inc., Waltham, MA), mouse anti-JAK2, mouse anti-phospho-JAK2 (Millipore), and mouse anti-beta-actin (Novus Biologicals). Blots were then probed with appropriate secondary horseradish peroxidase (HRP)-conjugated secondary antibodies (Jackson ImmunoResearch, West Grove, PA) and detected by an ECL detection system (Millipore). Data were analyzed using AlphaImager 2200 and densitometry analysis is expressed as ‘Fold induction’ normalized to β-actin.

### TG2 expression constructs and stable transfection

Full-length human TG2 was cloned into the pGene/V5-HisB vectors (Invitrogen, Carlsbad, CA, USA). RAW264.7 cells were transfected with the pSwitch vectors containing the mifepristone-inducible system, and then co-transfected with pGene/V5-HisB TG2 or pGene/V5-HisB vector alone using TurboFect (Fermentas, St. Leon-Rot, Germany) with several weeks of dual-select with zeocin and hygromycin B.

### siRNA treatment

siRNA was purchased from Santa Cruz Biotechnology. Transfection was performed with TurboFect (Fermentas) according to the manufacturer’s instructions. Cells were then treated with MSU crystals for an additional 24 h. Supernatants were collected for measurements of IL-1β, TNF-α and TGF-β1 concentration using ELISA.

### *In vivo* peritonitis induction

In the mouse model, we followed the protocol of a previous study [27]. Wild-type and TG2-null mice fully backcrossed onto BL57/6 background [31] were injected intraperitoneally with 1 ml PBS containing 3 mg MSU crystals (prepared as described earlier). During the final preparations the MSU crystals were washed in PBS. Injection of PBS alone had no effect on the cytokine formation or the neutrophil cell number injected into wild-type mice. Mice were sacrificed at the indicated time points and peritoneal cells were obtained by lavage with 2 ml sterile PBS. Total cell numbers were counted and percentage of neutrophil cells was determined by labeling the cells with fluorescein isothiocyanate (FITC)-conjugated anti-GR1 (BD Biosciences) antibody. Samples were analyzed on Becton Dickinson FACScan platform. Active TGF-β1 and IL-1β were determined from centrifuged, cell-free peritoneal lavage fluid by ELISA (eBioscience and R&D Systems, respectively). Mice were maintained in specific-pathogen-free condition in the Central Animal Facility and all animal experiments were approved by the Animal Care and Use Committee of University of Debrecen (DEMÁB).

### Statistics

The data were analyzed with GraphPad Prism 4 software by one-way analysis of variance (ANOVA) or Student’s *t*-test to determine the significance of differences between sets of categorical data. Data reported were verified in at least three different experiments and are represented as the mean ± standard error of the mean (SEM). A *P*-value <0.05 was considered to be significant.

## Results

### TG2 expression is enhanced in synovial fluid mononuclear cells of patients with GA and in MSU crystal-treated human PBMCs and monocyte-derived macrophages

**Figure 1:**
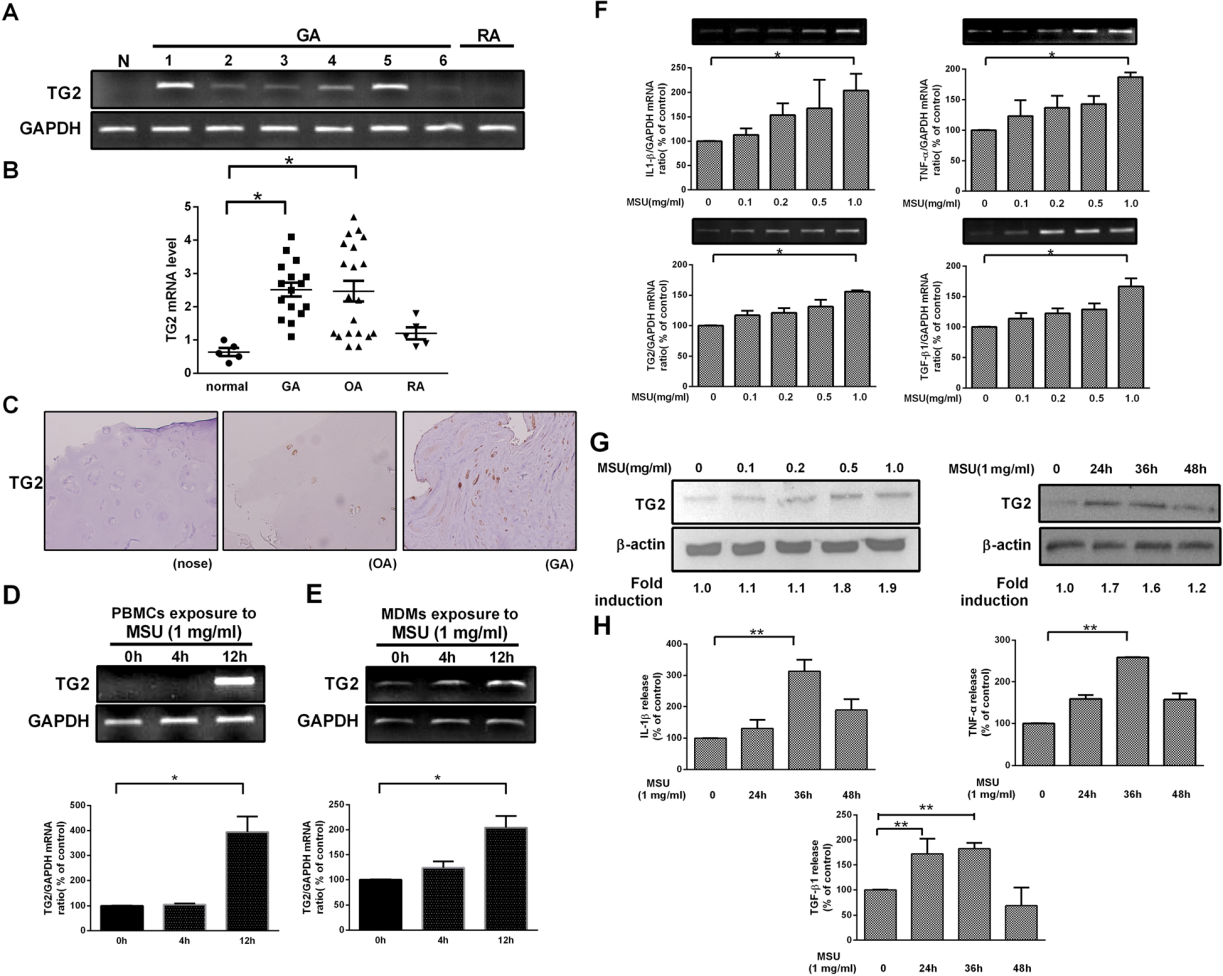
**Exposure to monosodium urate (MSU) crystals induces the expression of transglutaminase 2 (TG2) in synovial tissues of patients with gouty arthritis (GA).** **(A)** RT-PCR analysis of TG2 mRNA expression in synovial fluid mononuclear cells (SFMCs) from six patients with GA, one patient with rheumatoid arthritis (RA) and one with normal PBMCs (N). **(B)** mRNA levels of TG2 in SFMCs of patients with gouty arthritis (GA) (n = 16), osteoarthritis (OA) (n = 20), rheumatoid arthritis (RA) (n = 5) and in PBMCs of healthy donors (normal) (n = 5). Each point represents an individual patient. TG2 mRNA levels are expressed in arbitrary units (horizontal lines denote median values). **(C)** Representative images of immunohistochemical staining of TG2 in healthy tissue (left panel), osteoarthritis (OA) (middle panel) and GA (right panel) synovial tissues. Strong staining for TG2 was observed in the inflammatory cell infiltrate in GA synovium (Original magnification × 200). **(D-E)** PBMCs **(D)** and MDMs **(E)** isolated from healthy donors were stimulated with or without MSU crystals (1 mg/ml) for 0-12 h, and TG2 mRNA expression was analyzed by RT-PCR. **(F)** Induction of IL-1β, TNF-α, TG2 and TGF-β1 mRNA expression in RAW264.7 cells stimulated with MSU crystals (0.1-1.0 mg/ml) for 4 h analyzed by RT-PCR. **(G)** Western blot analysis of TG2 expression in whole-cell lysates from RAW264.7 cells stimulated for 24 h with increasing concentrations MSU crystals (0.1-1.0 mg/ml) and for the indicated time periods with 1 mg/ml MSU crystals. The ‘Fold’ induction was measured by densitometry analysis and normalized to β-actin. **(H)** Cells were stimulated with or without MSU crystals (1 mg/ml). The levels of IL-1β, TNF-α and TGF-β1 in supernatants were evaluated by ELISA at the indicated time points. The results are expressed as the means ± SEM from three independent experiments (n ≧ 3, * = *P* < 0.05, ** = *P* < 0.01 compared with control (100%)).

To investigate whether TG2 mRNA expression is altered in human GA, we examined synovial tissues of patients with GA by RT-PCR. Figure 1A demonstrates enhanced TG2 mRNA expressions in 6 randomly selected SFMCs from 16 patients with GA, as compared to that of healthy donors or rheumatoid arthritis patients. Details on TG2 expression in SFMCs from healthy donors and patients with GA, osteoarthritis or rheumatoid arthritis are given in Figure 1B. Our data confirm previous observations for the enhanced expression of TG2 in SFMCs from osteoarthritis patients [32] and demonstrate for the first time the enhanced expression of TG2 in gout as well. No correlation between the enhanced TG2 levels and the WBC counts were found (data not shown). Next, we further compared TG2 expression in the synovium of patients suffering from either GA or osteoarthritis (Figure 1C). Immunohistochemistry revealed that TG2 expression was present in the synovial tissue sections of both types of patient; however, an overall and stronger TG2 staining was detected in the GA synovium. In addition, in freshly isolated PBMCs and MDMs, exposure to MSU crystals for 12 h resulted in a strong induction of TG2 (Figure 1D, E). These results indicate that there is a higher TG2 expression in GA, and MSU crystals induce TG2 mRNA levels in PBMCs and MDMs *in vitro*.

### Exposure to MSU crystals induces the expression of both pro- and anti-inflammatory cytokines and that of TG2 in RAW264.7 macrophages

In order to examine whether TG2, pro- and anti-inflammatory cytokines were concomitantly induced in MSU crystal-induced acute gout inflammation, the mRNA expression levels of IL-1β, TNF-α, TG2 and TGF-β1 were determined by RT-PCR in RAW264.7 macrophages stimulated with different doses of MSU crystals for 4 h. We found that mRNA levels of IL-1β, TNF-α and TG2 were upregulated dose-dependently. TGF-β1 mRNA was modestly increased by MSU crystals at a dose as low as 0.1 mg/ml and was further upregulated between 0.2 and 1.0 mg/ml (Figure 1F). TG2 protein levels were also increased dose-dependently after MSU crystal exposure for 24 h (Figure 1G). Further, we analyzed the kinetics of TG2, IL-1β, TNF-α and TGF-β1 production induced by MSU crystals. As shown in Figure 1G, H, the amount of TG2 detected in the macrophages and that of IL-1β, TNF-α and TGF-β1 detected in the cell culture supernatants was increased following 24 to 36 h after stimulation, and decreased by 48 h. These data demonstrate that exposure to MSU crystals concomitantly increases the expression of IL-1β, TNF-α, TG2 and TGF-β1 in macrophages, TG2 and TGF-β1 being induced first.

### Knockdown or overexpression of TG2 inversely influences MSU crystal-induced IL-1β, TNF-α and TGF-β1 production in RAW264.7 cells

**Figure 2:**
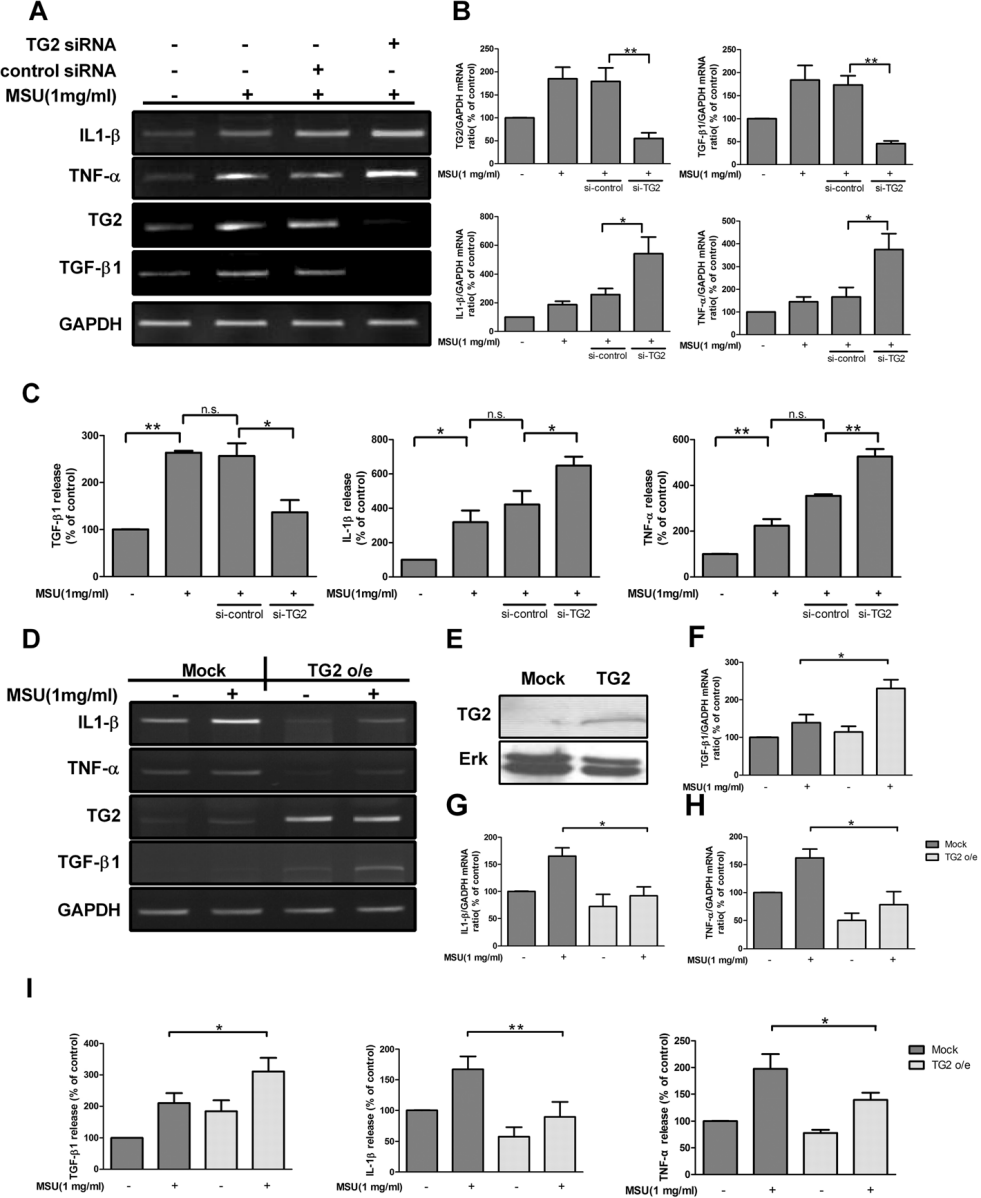
**Inverse effect of transglutaminase 2 (TG2) knockdown or overexpression on monosodium urate (MSU) crystal-induced cytokines production in RAW264.7 cells.** **(A)** RT-PCR analysis of IL-1β, TNF-α, TG2 and TGF-β1 mRNA expression in RAW264.7 cells transfected first with TG2 specific small interfering RNAs (siRNAs) for 48 h, then stimulated with or without MSU crystals (1 mg/ml) for 4 h. **(B)** Quantification of RT-PCR analysis of TG2, TGF-β1, IL-1β and TNF-α mRNA expressions. **(C)** Cells transfected with TG2-siRNA were stimulated with or without MSU crystals (1 mg/ml) for 36 h. The levels of TGF-β1, IL-1β and TNF-α in supernatants were collected and evaluated by ELISA. Mean control value of TGF-β1 (209.5 ± 21.2 pg/ml), IL-1β (23.1 ± 2.1 pg/ml) and TNF-α (137.8 ± 15.2 pg/ml). **(D)** RT-PCR analysis of IL-1β, TNF-α, TG2 and TGF-β1 mRNA expression in RAW264.7 cells stably transfected with TG2 or empty vector (mock) stimulated with or without MSU crystals (1 mg/ml) for 4 h. **(E)** Immunoblot analysis of TG2 in RAW264.7 cells stably transfected with TG2 or empty vector (mock). **(F-H)** Quantification of RT-PCR analysis of **(F)** TGF-β1, **(G)** IL-1β and **(H)** TNF-α mRNA expression. **(I)** Cells transfected with TG2 or empty vector were stimulated with or without MSU crystals (1 mg/ml) for 36 h. The levels of TGF-β1, IL-1β and TNF-α in supernatants were collected and evaluated by ELISA. The results are expressed as the means ± SEM from three independent experiments (n ≧ 3, * = *P* < 0.05, ** = *P* < 0.01 compared with control (100%)).

To investigate a possible influence of TG2 on cytokine regulation in acute gouty inflammation, we performed siRNA-mediated knockdown of TG2 in RAW264.7 cells. After 48 h post-transfection, cells were stimulated with MSU crystals for 4 h. As shown in Figure 2A and B, reduction in the endogenous TG2 expression by specific siRNA in RAW264.7 cells compromised the ability of MSU crystals to induce the expression of TGF-β1 mRNA, whereas MSU crystal-induced IL-1β and TNF-α mRNA expression was enhanced as compared to non-silencing control siRNA-treated cells. In accordance with the mRNA expressions, enhanced levels of pro-inflammatory cytokines, but reduced levels of TGF-β1, were detected at 36 h in the cell culture supernatants of the TG2 silenced cells in response to MSU crystals (Figure 2C).

Conversely, we generated RAW264.7 cell clones stably expressing TG2 or an empty vector (mock). RT-PCR analysis and immunoblotting were used to confirm overexpression of TG2 (Figure 2D-H). We found that MSU crystals (1 mg/ml) in TG2-overexpressing RAW264.7 cells induced higher TGF-β1 mRNA expression than in mock treated cells (Figure 2D and F). In contrast, TG2 overexpression attenuated MSU crystal-induced IL-1β and TNF-α mRNA levels (Figure 2D, G and H). In harmony with the mRNA expressions, MSU crystal-induced TGF-β1 levels were enhanced, whereas IL-1β and TNF-α amounts were downregulated in culture supernatants of TG2-overexpresseing RAW264.7 cells (Figure 2I). These results indicate that TG2 negatively and positively influences the MSU crystal-induced pro- and anti-inflammatory cytokine production, respectively.

### Effect of TG2 knockout on MSU crystal-induced IL-1β and TGF-β1 production in MEF cells

**Figure 3:**
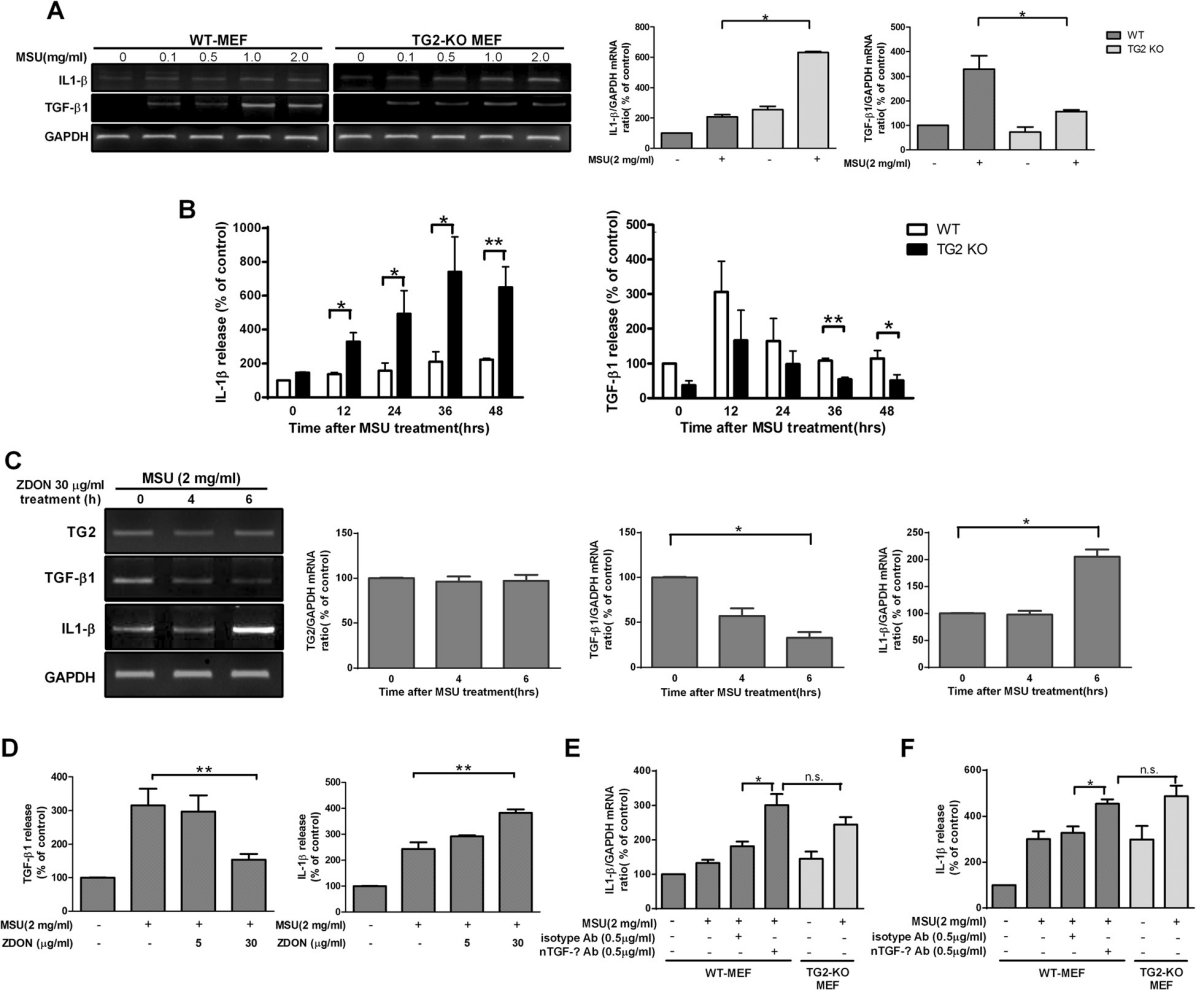
**Cross-linking activity of transglutaminase 2 (TG2) attenuates monosodium urate (MSU) crystal-induced production of IL-1β.** **(A)** RT-PCR analysis of IL-1β and TGF-β1 mRNA expression in WT and TG2-KO MEF cells stimulated with or without MSU crystals (0.1-2.0 mg/ml) for 4 h. Quantification of RT-PCR analysis of IL-1β and TGF-β1 mRNA expression is also shown. **(B)** Cells were stimulated with or without MSU crystals (2 mg/ml). The levels of IL-1β and TGF-β1 in supernatants were evaluated by ELISA at the indicated time points. **(C)** RT-PCR analysis of TG2, TGF-β1 and IL-1β mRNA expression in WT MEF cells stimulated with 2 mg/ml MSU crystals for the indicated time points in the presence of 30 μg/ml ZDON, an active-site inhibitor of TG2. Quantification of RT-PCR analysis of TG2, TGF-β1 and IL-1β mRNA expressions is also shown. **(D)** Cells were stimulated with MSU crystals (2 mg/ml) for 36 h in the presence of increasing concentrations of ZDON. The levels of TGF-β1 and IL-1β in supernatants were evaluated by ELISA. Mean control value of TGF-β1 (278.3 ± 18.2 pg/ml) and IL-1β (39.4 ± 5.9 pg/ml). **(E)** Quantification of RT-PCR analysis for IL-1β expression in WT and TG2-KO MEF cells stimulated with or without MSU crystals (2 mg/ml) for 4 h in the presence of increasing amounts of neutralizing anti-TGF-β1 or its isotype control antibody. **(F)** MEF cells were stimulated with MSU crystals (2 mg/ml) for 36 h in the presence and absence of neutralizing TGF-β antibody. IL-1β in supernatants was evaluated by ELISA. The results are expressed as the means ± SEM from three independent experiments (n ≧ 3, * = *P* < 0.05, ** = *P* < 0.01 compared with control (100%)).

As siRNA-mediated knockdown studies are dependent on the extent of target knockdown, we decided to validate the above findings by using genetically TG2-depleted MEF cells [33]. We found that TG2 deficiency substantially enhanced the ability of MSU crystals to induce IL-1β mRNA, but it suppressed the MSU crystal-induced levels of TGF-β1 mRNA in MEF cells as well (Figure 3A). A time-course study revealed that exposure of MEF cells to MSU crystals (2 mg/ml) enhanced IL-1β secretion, with a maximal increase at 36 h, while that of TGF-β1 had already occurred by 12 h. While TG2 deficiency resulted in decreased MSU crystal-induced secretion of TGF-β1, it enhanced that of IL-β (Figure 3B). These findings indicate that TG2 might play a pivotal role in the self-limitation of MSU crystal-induced pro-inflammatory cytokine formation.

### Cross-linking activity of TG2 is required for promoting and attenuating the MSU crystal-induced TGF-β1 and IL-1β production, respectively

TG2 is a multifunctional protein. To decide whether its protein cross-linking or other biological activities are required for altering the MSU crystal-induced cytokine formation, wild-type MEF cells were pretreated with or without the cell-permeable TG2 active-site inhibitor ZDON for 4 h or 6 h. Then the cells were stimulated with MSU crystals for 4 h. As shown in Figure 3C, blockade of TG2 cross-linking activity abolished TGF-β1 mRNA expression after MSU crystals stimulation. In harmony with the mRNA results, pretreatment with ZDON also attenuated the MSU crystal-induced TGF-β1 release, while it promoted that of IL-1β (Figure 3D). These data indicate that TG2 influences MSU crystal-induced cytokine release by using its protein cross-linking activity.

### MSU crystal-induced TGF-β1 activity negatively regulates MSU crystal-induced IL-1β production

To investigate whether the observed enhanced MSU crystal-induced IL-1β production in TG2-null MEF cells could be related to an inefficiently functioning TG2-TGF-β1 pathway, we neutralized the activity of TGF-β1 by a pan TGF-β neutralizing antibody. Application of the neutralizing antibody applied in concentrations of 0.5 to 5.0 μg/ml resulted in enhancement of MSU crystal-induced IL-1β mRNA expression (Figure 3E and Additional file 2) and IL-1β production (Figure 3F) in wild-type MEF cells without affecting the MSU crystal-triggered TGF-β1 induction providing evidence for negative regulation of MSU crystal-induced IL-1β production by the MSU crystal-induced TGF-β1 signaling pathway.

### MTA1 is required for the MSU crystal-induced TG2 and TGF-β1 expressions

**Figure 4:**
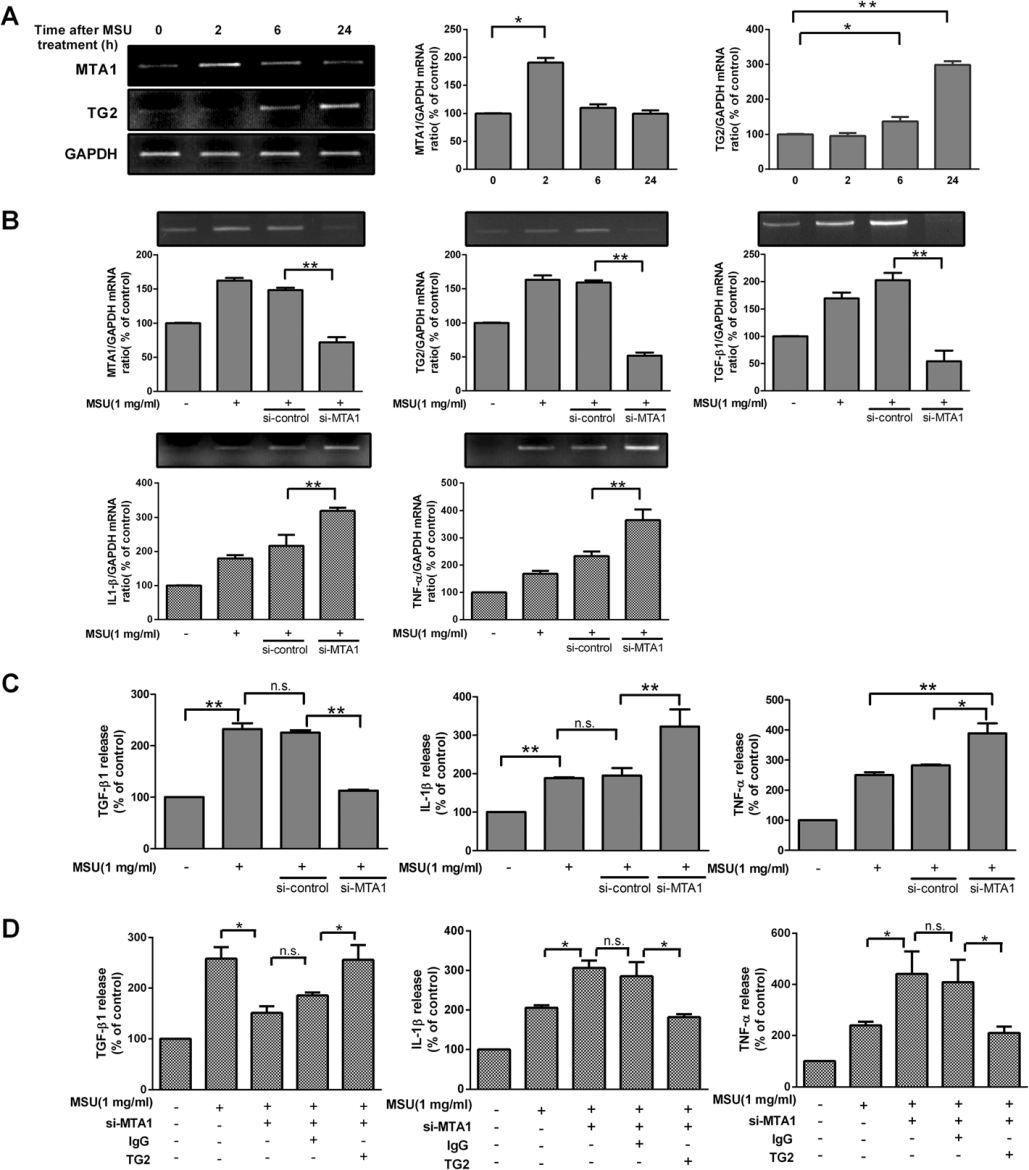
**Effect of metastatic tumor antigen 1 (MTA1) knockdown on monosodium urate (MSU) crystal-induced cytokine production in RAW264.7 cells.** **(A)** Time course of the MSU crystal (1 mg/ml) induced increase in MTA1 and TG2 mRNA expression in RAW264.7 detected by RT-PCR. Quantification of RT-PCR analysis of MTA1 and TG2 mRNA expressions is also shown. **(B)** RT-PCR analysis of RAW264.7 cells transfected with MTA1 specific small interfering RNAs (siRNAs) for 48 h and stimulated then with or without MSU crystals (1 mg/ml) for 4 h. Quantification of RT-PCR analysis of MTA1, TG2, TGF-β1, IL-1β and TNF-α mRNA expressions. **(C)** Cells were stimulated with or without MSU crystals (1 mg/ml) for 36 h. The levels of TGF-β1, IL-1β and TNF-α in supernatants were collected and evaluated by ELISA. **(D)** In addition, MTA1 knockdown macrophages were also treated by MSU crystals (1 mg/ml) in the presence of recombinant TG2 (10 ng/ml) or IgG (10 ng/ml) for 36 h. The levels of TGF-β1, IL-1β and TNF-α in supernatants were collected and evaluated by ELISA. The results are expressed as the means ± SEM from three independent experiments (n ≧ 3, * = *P* < 0.05, ** = *P* < 0.01 compared with control (100%)).

As MTA1 is known to act as a co-regulator in the basal and lipopolysaccharide-induced expression of TG2 in macrophages [24], we decided to determine whether the MTA1-TG2 axis is also involved in the negative feedback regulation of MSU crystal-induced inflammation. First we analyzed the alterations in MTA1 and TG2 mRNA expression in RAW264.7 cells stimulated with MSU crystals. An early induction of MTA1 mRNA was observed in RAW264.7 cells by 2 h in response to MSU crystals, which preceded the induction of TG2 mRNA (Figure 4A). To decide whether MTA1 contributes to MSU crystal-induced TG2 expression, we performed knockdown of MTA1 by siRNA in RAW264.7 cells (Figure 4B). Silencing of MTA1 by specific siRNA in RAW264.7 cells compromised the ability of MSU crystals to induce the expression of TG2 and TGF-β1 mRNA and protein. Conversely, the production of IL-1β and TNF-α was increased (Figure 4B, C). To prove that the observed effects of MTA1 silencing are indeed related to the prevention of TG2 expression, recombinant TG2 was added to MTA1 knockdown cells. As shown in Figure 4D, addition of recombinant TG2 was able to correct the defect in TGF-β1 production leading to a decrease in the MSU-induced IL-1β and TNF-α production in MTA1-silenced cells. Our findings indicate that MTA1 offers protection against MSU crystal-induced inflammation by directly enhancing the TG2-dependent TGF-β1 signaling.

### Loss of TG2 enhances the MSU crystal-induced production of IL-1β and decreases that of TGF-β1 in an *in vivo* peritonitis model

**Figure 5:**
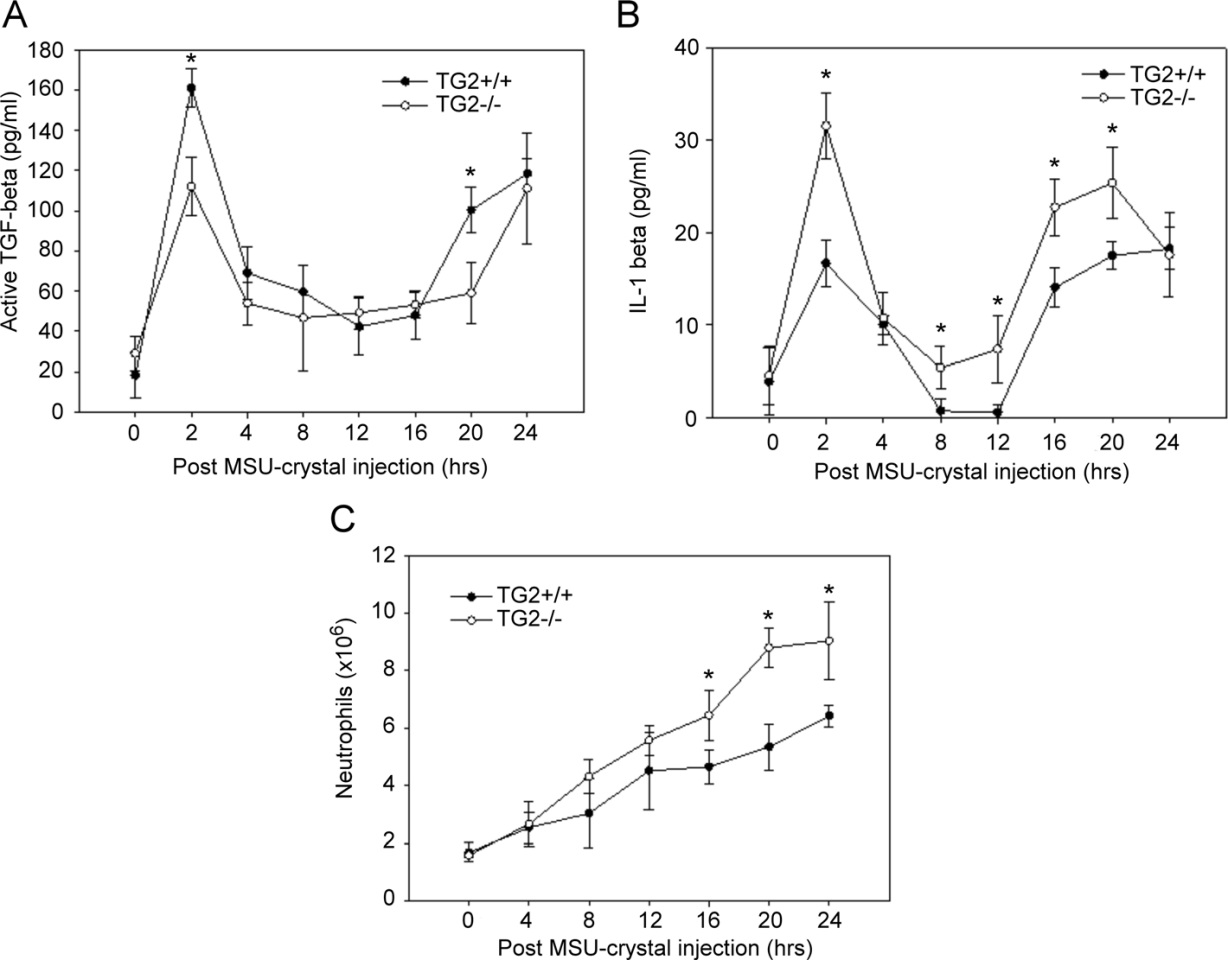
**Effect of transglutaminase 2 (TG2) deficiency on monosodium urate (MSU) crystal-induced induced IL-1β and transforming growth factor (TGF)-β1 production** ***in vivo*** **.** Wild-type (WT) and TG2-deficient mice were injected intraperitoneally with 3 mg MSU crystals. Mice were sacrificed at the indicated time points and peritoneal cells were obtained by lavage with 2 ml sterile PBS. **(A,** **B)** The levels of active TGF-β1 **(A)** and IL-1β **(B)** were determined at the indicated time points in the centrifuged, cell-free peritoneal lavage fluids, by ELISA. **(C)** The numbers of Gr-1^hi^ neutrophils in the peritoneum were analyzed by flow cytometry. The experiments were repeated three times. In each experiment three mice were used at each time point. The results are expressed as the mean ± SEM from three independent experiments (n ≧3, *P <0.05 and **P <0.01).

To study the influence of TG2 on MSU crystal-induced inflammation *in vivo*, MSU crystals were injected into the peritoneum of wild-type and TG2-null mice [31] to examine the time-dependent cytokine formation and neutrophil immigration following MSU crystal exposure. Injection of 3 mg MSU crystals into the peritoneum resulted in a biphasic increase in both the IL-1β and in the active TGF-β1 production, the first peak appearing at around 2 h, while the second was at around 20 h following MSU crystals injection. Loss of TG2 did not affect the early kinetics of cytokine formation. However, as compared to the wild-type animals, the second peak of TGF-β1 release was delayed in the TG2-null animals. In addition, the expression of the pro-inflammatory cytokine IL-1β was increased, while that of the anti-inflammatory cytokine TGF-β1 was decreased in TG2-null mice (Figure 5A, B). Moreover, in the peritoneal cavity of TG2-null animals a significantly increased neutrophil number was detected upon treatment, demonstrating the negative regulatory role of TG2 in the MSU crystal-induced inflammation also in the intact organism (Figure 5C).

### Effects of TG2 overexpression and knockout on MSU crystal-induced JAK2 phosphorylation

**Figure 6:**
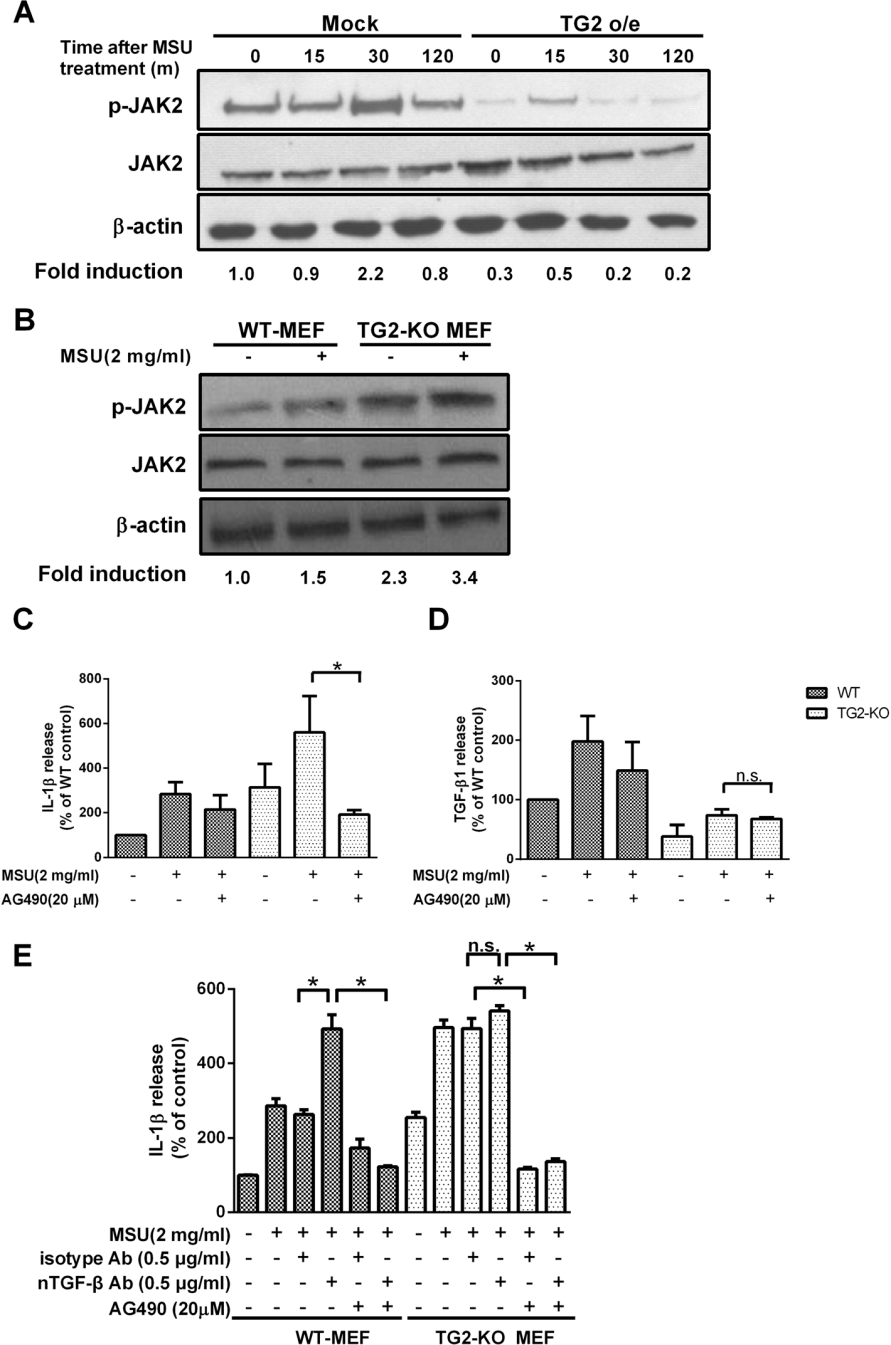
**Transglutaminase 2 (TG2) inhibits the basal and the monosodium urate (MSU) crystal-induced JAK2 activation.** **(A)** RAW264.7 cells stably transfected with TG2 or empty vector (mock) were stimulated with or without MSU crystals (1 mg/ml) for the indicated time points. Whole-cell lysates were analyzed by western blot analysis using antibodies against JAK2, phospho-JAK2 and β-actin. The ‘Fold’ induction was measured by densitometry analysis and normalized to β-actin. **(B)** Wild-type and TG2 null MEF cells were treated for 1 h with or without MSU crystals (2.0 mg/ml). Whole-cell lysates were analyzed by western blot analysis using antibodies against JAK2, phospho-JAK2 and β-actin. β-actin was used as a loading control. The ‘Fold’ induction was measured by densitometry analysis and normalized to β-actin. **(C-D)** Wild-type and TG2 null MEF cells were treated for 36 h with or without MSU crystals (2.0 mg/ml) or in the presence of the JAK2 inhibitor (AG490). **(C)** IL-1β and **(D)** TGF-β1 in the supernatants were evaluated by ELISA. **(E)** Experiments described in **C** were repeated in the presence of 0.5 g/ml neutralizing anti-TGF-β antibody and its isotype control. The results are expressed as the means ± SEM from three independent experiments (n ≧ 3, * = *P* < 0.05 compared with control (100%)).

A recent study carried out in RAW264.7 macrophages indicated that a JAK2-STAT3, JAK2-AKT-STAT3 pathway can regulate IL-1β expression in these cells [34]. Thus, we determined basal and MSU crystal-induced JAK2 (Tyr1007/Tyr1008) phosphorylation levels to decide whether loss of TG2 has any effect on it. As shown in Figure 6A and B, increasing the level of TG2 in RAW264.7 macrophages decreased, while loss of TG2 in MEF cells increased the basal level of phosphorylation of JAK2, which correlates with its kinase activity. In addition, exposure to MSU crystals increased the phosphorylation level of JAK2 in both macrophages and MEF cells in a time-dependent manner (Figure 6A, B).

To determine whether p-JAK2 participates in the MSU crystal-induced IL-1β production, MEF cells were pretreated with the JAK2-specific inhibitor AG490 for 60 minutes and then exposed to MSU crystals. As shown in Figure 6C, the enhanced MSU crystal-induced IL-1β production in TG2-null macrophages was completely diminished by AG490, while it only slightly attenuated the MSU crystal-induced IL-1β production in wild-type cells. In contrast, the TGF-β1 production was not affected by JAK2 inhibition in any of the cells (Figure 6D) indicating that the effect of JAK2 inhibition on the IL-1β production was not mediated by regulation of TGF-β1 levels. As shown in Figure 6E, administration of neutralizing TGF-β1 antibodies enhanced the MSU crystal-induced IL-1β production in wild-type MEFs, and this enhancement was completely prevented by AG490, while administration of neutralizing TGF-β antibodies did not affect the MSU crystal-induced response of TG2 null MEFs. These data provide evidence that TG2-induced TGF-β1 suppresses the MSU crystal-induced IL-1β production by interfering with the MSU crystal-induced JAK2 signaling pathway.

## Discussion

In this study, we provided evidence that TG2 contributes to the limitation of inflammation in GA. We found that TG2, as reported for IL-1β, TNF-α and TGF-β1 [15], is expressed in higher levels in SFMCs of patients with GA, and we observed that the protein is also highly expressed in GA synovial tissues. In line with these observations, we found that exposure to MSU crystals induces the expression of TG2 in human and mouse macrophages, as well as in mouse fibroblasts. The mechanism of MSU crystal-induced increase in TG2 expression in these cells was not investigated in our studies, but we demonstrated that similar to the lipopolysaccharide-induced increase in TG2 expression in macrophages, it required the induction of MTA1, a master co-activator acting on the chromatin.

Immune systems have evolved multiple strategies to regulate and maintain an adequate level of inflammation, including induction of negative feedback regulators for inflammation. TGF-β1 [12] has been suggested to play such a negative feedback regulatory role in gout by suppressing MSU crystal-induced acute inflammation *in vivo* [11,13]. In the present study we identified the MTA1-TG2 signaling pathway as a critical component for TGF-β1 production during MSU crystal-induced inflammation, as both silencing of MTA1 and TG2 resulted in a lower TGF-β1 production. TG2 very likely acts on the cell surface in this process, because its loss could be compensated by adding recombinant TG2 extracellularly to the cells. In addition, induction of active TGF-β1 by TG2 requires the cross-linking activity of the protein, because it was inhibited by the highly specific active-site inhibitor, ZDON. Interestingly, in a recent study it was also found that increased expression of TG2 leads to enhanced TGF-β1 mRNA levels in fibroblasts [35]. Induction of TGF-β1 mRNA by TG2 was a result of NF-κB activation, as the TGF-β1 promoter consists in NF-κB response elements [36]. Though in our studies the possible involvement of NF-κB in the MSU crystal-induced, TG2-dependent TGF-β1 induction was not investigated, it is worth noting that TG2 is able to activate NF-κB in various cell types acting either intra- or extracellularly. However, while intracellular TG2 activates NF-κB through an IκBα degradation-dependent mechanism [37], extracellular TG2 was reported to trigger the non-canonical pathway of NF-κB activation [38]. The receptors through which extracellular TG2 affects NF-κB activation have not been identified yet, but integrin receptors, for which TG2 acts as a co-receptor are known to activate the non-canonical NF-κB pathway [39].

We have also demonstrated that TGF-β1 induced by MSU-crystal exposure negatively regulates MSU crystal-induced pro-infammatory cytokine formation in macrophages, in a similar way as it does in MSU crystal-exposed neutrophils [40]. Our data also indicate that it does so by interfering with the MSU crystal-induced JAK2 signaling pathway. Based on our data we propose that MSU crystals trigger two independent signaling pathways in phagocytes, (1) the JAK2-STAT pathway, which leads to IL-1β production, and (2) the MTA1-TG2-TGF-β1 pathway, which negatively regulates the first. Though the mechanism through which this antagonism is mediated was not determined in these studies, all together our data provide evidence for the existence of an MTA1-regulated TG2-TGF-β1 axis that might act as a negative feedback regulatory pathway in MSU crystal-induced inflammation.

To extend our studies we have also investigated the consequence of the loss of TG2 on MSU crystal-induced IL-1β and TGF-β1 formation in an *in vivo* mouse peritonitis model by using TG2-null animals. We could confirm previous findings [27] that demonstrated that loss of TG2 enhances the number of neutrophils in the peritoneal cavity following exposure to MSU crystals. However, unlike in those studies, we found a significant difference in both the MSU crystal-induced TGF-β1 and IL-1β production. Injection of MSU crystals into the peritoneum resulted in a increase in both the IL-1β and in the active TGF-β1 release, but similar to the *in vitro* data the levels of active TGF-β1 were higher, while that of IL-1β were lower in wild-type mice as compared to TG2-null mice. As the loss of TG2 was reported to also affect the efficiency of apoptotic cell clearance [24-26], our data indicate that *in vivo* TG2 might limit GA acting via several different simultaneous mechanisms including enhancing efferocytosis and decreasing the production of pro-inflammatory cytokines.

## Conclusions

In summary, we describe for the first time that MTA1 plays an anti-inflammatory role in MSU crystal-induced inflammation. We propose that in MSU crystal-induced inflammation the MTA1-TG2 signaling pathway contributes to the resolution of inflammation by enhancing TGF-β1 production, which in turn attenuates MSU crystal-induced IL-1β and TNF-α production. Our data indicate that TG2 plays a determining role in maintaining the balance in the MSU crystal-induced pro- and anti-inflammatory cytokine production, provide further evidence for the anti-inflammatory role of TG2 in gout, and suggest that TG2 might have implications in the treatment of gouty arthritis.

## Additional files

Additional file 1:
**Primer sequences.** Primer sequences of the genes used for reverse transcriptase PCR (RT-PCR). Additional file 2:
**Effect of TGF-β neutralizing antibody on MSU crystal-induced IL-1β mRNA expression.** RT-PCR analysis of IL-1β, and TGF-β1 mRNA expressions in WT and TG2-KO MEF cells stimulated with or without MSU crystals (2.0 mg/ml) for 4 h in the presence of increasing amounts of neutralizing anti-TGF-β1.

## Abbreviations

DMEM: Dulbecco’s modified Eagles’s medium
ELISA: enzyme-linked immunosorbent assay
GA: gouty arthritis
GAPDH: glyceraldehyde-3-phosphate dehydrogenase
IL: interleukin
MDM: monocyte-derived macrophage
MEF: mouse embryonic fibroblast
MSU: monosodium urate
MTA1: metastatic tumor antigen 1
PBMC: peripheral blood mononuclear cell
PBS: phosphate-buffered saline
RT-PCR: reverse transcriptase PCR
SFMC: synovial fluid mononuclear cell
siRNA: small interfering RNA
ST: synovial tissue
TG2: transglutaminase 2
TGF-β1: transforming growth factor β1
TNF: tumor necrosis factor
WBC: white blood cell
