# Supplementary material for: The metastatic tumor antigen 1-transglutaminase-2 pathway is involved in self-limitation of monosodium urate crystal-induced inflammation by upregulating TGF-β1
Source: Arthritis Res Ther. 2015 Mar 19;17(1):65. doi: 10.1186/s13075-015-0592-7 (PMC4422600; doi:10.1186/s13075-015-0592-7)
Supplement: Additional file 1: — Primer sequences. Primer sequences of the genes used for reverse transcriptase PCR (RT-PCR). [file 13075_2015_592_MOESM1_ESM.pdf]

Additional file 1: Primer sequences

Primer sequences of the genes used for RT-PCR.

Mouse Gene	Forward	Reverse
IL-1β	5'- TCCATGAGCTTTGTACAAGGA -3'	5'- AGCCCATACTTTAGGAAGACA -3'
TNF-α	5'- ATGAGCACAGAAAGCATGATC -3'	5'- TACAGGCTTGTCACTCGAATT -3'
TGF-β	5'- CAACAATTCCTGGCGATACC -3'	5'- GAACCCGTTGATGTCCACTT -3'
TG2	5'- TGATGACCGGGAGGACATCA -3'	5'- GATTCTCCAGGTAGAGATCTC -3'
MTA1[22]	5'- AGTGCGCCTAATCCGTGGTG -3'	5'- CTGAGGATGAGAGCAGCTTTCG -3'
GAPDH	5′- TCACTCAAGATTGTCAGCAA -3′	5′- AGATCCACGACGGACACATT -3′

Human Gene	Forward	Reverse
TG2	5'-CTCGTGGAGCCAGTTATCAACAGCT-3'	5'- TCTCGAAGTTCACCACCAGCTTGTG -3'
GAPDH	5'- CATGTTCGTCATGGGTGTGA -3'	5'- AGTGAGCTTCCCGTTCAGCT -3'
