# Supplementary figures and images for: The metastatic tumor antigen 1-transglutaminase-2 pathway is involved in self-limitation of monosodium urate crystal-induced inflammation by upregulating TGF-β1
Source: Arthritis Res Ther. 2015 Mar 19;17(1):65. doi: 10.1186/s13075-015-0592-7 (PMC4422600; doi:10.1186/s13075-015-0592-7)

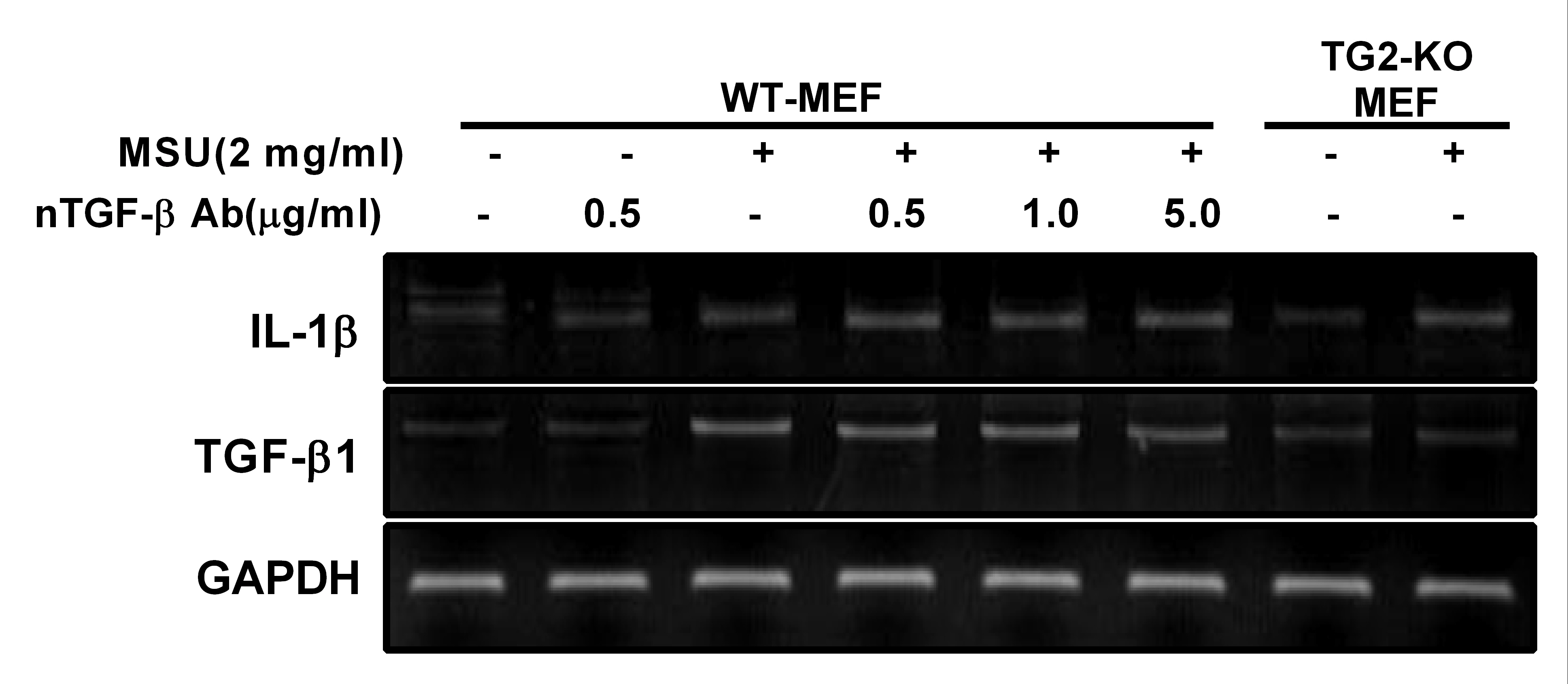

Supplement: Additional file 2: — Effect of TGF-β neutralizing antibody on MSU crystal-induced IL-1β mRNA expression. RT-PCR analysis of IL-1β, and TGF-β1 mRNA expressions in WT and TG2-KO MEF cells stimulated with or without MSU crystals (2.0 mg/ml) for 4 h in the presence of increasing amounts of neutralizing anti-TGF-β1. [file 13075_2015_592_MOESM2_ESM.jpeg]
